# Generation Gaps in Digital Health Literacy and Their Impact on Health Information Seeking Behavior and Health Empowerment in Hungary

**DOI:** 10.3389/fpubh.2021.635943

**Published:** 2021-05-13

**Authors:** Orsolya Papp-Zipernovszky, Mária Dóra Horváth, Peter J. Schulz, Márta Csabai

**Affiliations:** ^1^Department of Personality, Clinical and Health Psychology, Institute of Psychology, University of Szeged, Szeged, Hungary; ^2^Department of Health Economics, Faculty of Medicine, University of Szeged, Szeged, Hungary; ^3^Institute of Communication and Health, Faculty of Communication Science, University of Lugano, Lugano, Switzerland

**Keywords:** generations, internet health information seeking, eHealth literacy, eHeals, health empowerment

## Abstract

**Background:** Today the internet is a major source of health information, and younger generations have more confidence in their digital information seeking skills and awareness of online resources than older generations. Older generations, however, are more in demand of health services. The aim of our study was to explore these generational differences as related to self-perceived eHealth literacy and health care system utilization.

**Methods:** A cross-sectional survey study with 522 subjects was done in Hungary. Every subject belonged to one of four generations (Baby boomers, X, Y, and Z). The Web-based survey was designed and tested in English-speaking countries and translated into Hungarian for the present study. Variables include Internet health information seeking, eHealth literacy (measured by eHeals score), the self-perceived gain in empowerment by that information, and the number of health care appointments. One-way ANOVA was used for comparing the scores of the generations, and correlational and linear regression analysis was employed within the generations for further data analysis.

**Results:** We found significant differences among the generations in eHealth literacy as well as in the self-perceived gain in empowerment: while Boomers were the generation with the lowest eHeals scores, they showed the highest empowerment. Internet health information seeking behavior showed no differences. While subjects who use the Internet more frequently to search for health information have worse self-rated health status, the ones with higher eHeals scores report better subjective health status. We also identified the associations of the above variables within the older generations (Boomers and X) with the frequency of using health-care services: within the generation of Boomers the number of health care appointments was only associated with Internet health information seeking, while in Generation X with eHeals.

**Conclusions:** Baby boomers seek Internet health information as often as the younger generations, which provides a solid motivation for developing their eHealth literacy skills. We find it crucial to plan the Hungarian health promotion programmes utilizing this high frequency of Internet health information seeking, since the eHealth literacy skills of older generations have an effect on their subjective health status, and they are the most capable of applying information in making decisions.

## Introduction

Reviewing the health literacy (HL) literature, Martensson and Hensing (1) found that in one strain of definitions the complexity of HL is stressed due to its dynamic nature, the multidimensional interrelations it keeps, and the embeddedness in a social or cultural context. In research it includes the interactive and critical type of HL (2), which deals with the contexts other than health institutions where health-related information is collected from (e.g., the Internet) as well as with the ways and actions this information is used. In the present paper we follow the social-ecological model of HL by examining a specific context of health information seeking and the related skills, namely the Internet. Furthermore, we attempt to reveal generational differences as a social phenomenon underlying health information seeking and eHealth.

eHealth is defined as “the use of information and communication technology (ICT) for health” (3). Gilstad (4) established eHL as “the ability to identify and define a health problem, to communicate, seek, understand, appraise and apply eHealth information and welfare technologies in the cultural, social and situational frame and to use the knowledge critically in order to solve the health problem.”(p. 69). Generational differences shown in eHealth could also be interpreted within this framework.

According to McCrindle and Wolfinger (5) generations are groups of individuals who live in the same period and are influenced by the same technologies and experiences. The generational differences in social characteristics may result in variations in one's ability to employ technologies (6, 7), to use diverse strategies for health information seeking on the Web (IHISB) and to show different levels of digital HL. Furthermore, the literature also shows generational differences regarding health in relation to changes in lifestyle, and to health status (8). Being aware of these generational differences may be of great importance in forming health policy decisions and the health care market. Finally, we include self-perceived gain in empowerment from using Internet health information as a variable to tap into the application of the information and another variable, the utilization of the healthcare system that is related to the institutional aspects of HL.

### Internet Health Information Seeking Behavior

The internet seems to be the most popular source of health information (9). Andreassen et al. (10) found that among European citizens, 71% of Internet users utilize the Internet for health purposes. They also reported that young age, higher education, white-collar or no paid job, number of visits to the general practitioner during the past year, long-term illness or disabilities and good subjective health assessment are positively affecting the use of the Internet for health purposes. According to more recent data, published in 2020 by Eurostat, 55% of individuals used the internet for seeking health information in 2020 within the 27 European countries (11). Specifically in Hungary 63% of individuals used the internet for health information seeking (11).

Jiang and Street (12) studied the health outcomes of Internet health information seeking behavior (IHISB) by testing a moderated mediation pathway model based on the three-stage model of health promotion (13, 14). According to their findings, IHISB affects general physical and emotional health outcomes. This effect is mediated by the access to social support resources, which is positively moderated by users' online health information seeking experience (12). Whether IHISB improves the patient-physician relationship (15) depends on the previous quality of the relationship as well as on whether patients discuss information they've accessed online.

### eHealth Literacy

Some aspects of IHISB, such as its frequency and the kind of sources it relies on, have proved to be an indirect measurement of eHL (16, 17). According to Norman and Skinner eHL is “the ability to seek, find, understand, and appraise health information from electronic sources and apply the knowledge gained to addressing or solving a health problem” (18). As proposed in their Lily model, eHealth literacy consists of three contextual literacies (health literacy, computer literacy, and science literacy) and three analytical literacies (traditional literacy, information literacy, and media literacy) (18). Gilstad supplemented this model with the acknowledgment of the bodily experience of a health challenge, the procedural literacy of handling the tools and technologies, the contextual and the cultural literacy and the communicative expertise (4).

Noorgard et al. (19) introduced the newest eHealth Literacy Framework, consisting of seven dimensions. They used concept mapping involving patients, health professionals and medical informatics experts to generate their model. The following domains of eHL were identified: “Ability to process information,” “Engagement in own health,” “Ability to engage actively with digital services,” “Feeling safe and in control,” “Motivation to engage with digital services,” “Having access to systems that work,” and “Digital services that suit individual needs.” Their framework provides insights into one's ability to understand, access and use e-health technologies (19).

Although, according to Neter and Brainin (20), research on the effects of eHL on health outcomes is still in its early stage, Diviani et al. (21) found that eHL is positively associated with the ability to evaluate and trust online health information. Furthermore, the higher the level of eHL is for an individual, the better health outcomes (e.g., better self-rated health status) (22, 23) they may achieve, through better communication with their physician, practicing more health behaviors (e.g., higher likelihood of undergoing cancer screening, eating a balanced diet or doing physical exercise) (24, 25), better understanding of their condition, and increased use of medical insurance (20, 26, 27).

Understanding the sociodemographic context of IHISB and eHL is becoming increasingly significant as the Internet becomes the major source of health information seeking. Age particularly is a major factor that influences both eHL (28–30) and IHISB (31, 32). However, validating the Hungarian eHeals scale Zrubka et al. (17) reported only a significant negative but weak correlation between age and eHeals scores. They found that being over 65 years of age is a risk factor in lacking an appropriate level of digital HL, which is in accordance with our previous results considering the level of functional HL in the Hungarian population (33). In our attempt of measuring eHeals and its associations we rely on a more complex age-based comparison, namely, generational differences.

### Generations

A generation is defined by a birth period of 20–25 years, in other words as long as it takes for the group to be born, grow up and have children (34, 35). The generations might have common attitudes, values and beliefs as they were born in the same period and lived through similar experiences of social, political and economic events during their youth (35). McCrindle and Wolfinger (5) distinguish seven categories of generations by year of birth: Federation Generation (1901–1924), Builders (1925–1945), Baby Boomers (1946–1964), Generation X (1965–1979), Generation Y (1980–1994), Generation Z (1995–2009) and Generation Alpha (2010–). In the following we summarize the attitude and skills toward technology and health needs of the four generations who participated in our research.

#### Baby Boomers (1946–1964)

The generation of baby boomers were born after the Second World War (36). Boomers were young when computerized systems became a part of everyday life. As they are an aging generation, health is an important issue for them (37). While they maintain a higher awareness in certain consumption choices, including bodily maintenance, diet, and exercise (38) and are more willing to take a greater role in their own health care, they are not particularly healthy (24).

Baby boomers and the previous generation most commonly use their electronic devices to seek internet health information (39). Medlock et al. (40) examined which information resources the Internet-using seniors (67–78 years) turn to and trust for health information. The most commonly used and trusted health information sources were health professionals, pharmacists and the Internet. The higher use of the Internet was associated with higher use of other sources. Participants used diverse sources for different types of information. The Internet was most often used for seeking information about symptoms, prognosis and treatment options, whereas health professionals were asked for information on medications, side effects, coping, practical care and nutrition or exercise.

HL seems to deteriorate with aging, and lower HL has a negative impact on health care access, chronic disease management and health status (41, 42). These also come with increased health care costs, more medication errors, ineffective patient-provider communication and inefficient use of health care services (43). According to the findings of Choi and Dinitto (44), eHL is also negatively associated with age. Tennant et al. (45) examined the relationship between sociodemographic variables, the use of electronic devices and Web 2.0. for health information and eHL among baby boomers and older adults (being 50 years of age or over). They found that within this population younger age, higher education, use of more electronic devices and the use of Web 2.0 platforms are associated with higher levels of eHL. The direct antecedent of our research was conducted by Schulz et al. (37) focusing on the relationships between IHISB, eHL and specific health outcomes, i.e., the number of consultations with one's GP and self-rated health status among anglo-saxon baby boomers. They found moderate relationships between IHISB, eHL and perceived gain in empowerment, while there was no direct association between eHL and utilization of the health care system, but indirect effect paths via the former variables.

#### Generation X (1965–1979)

Individuals belonging to generation X had to grow up in economic uncertainty due to the recessions of the early 1980's and 1990's. Societal uncertainty was also a general fact due to the increase of divorces or both parents working (46, 47). Hence the majority of this generation became independent at a young age (48). The technical ability of this generation tends to be strong (49, 50). They were the first generation to grow up when the Internet started to make health information available (51). They rely on technology (52) and social media (53) very much when it comes to their healthcare needs (51). Seventy-four percentage of them said in a research that they would rather visit the doctor through telemedicine than in person (52).

They are more skeptical toward healthcare systems compared to preceding generations and they prefer doctors as a source of information about health (54). They trust their physicians more than the generation Y (55). They are motivated to look for information in numerous sources such as: family members, co-workers, doctors, pharmaceutical company websites, medical journals, news websites and books (51).

#### Generation Y (1980–1994)

The Y generation grew up in a period of economic growth (56). The individuals in this generation cohort are technologically competent (57, 58) as they manage their lives and daily activities with the help of digital technologies (48). They are referred to as “the first generation of digital natives” (59). According to Kim and Son (25), the main source of health information for 18–39-year-old adults is the Internet. eHL was found to be associated with patterns of health behaviors in this generation. Bianca Mitu (60) also reported that 18–31-year-old people with medium or high eHL use more than one source of information and a variety of online search strategies. The majority of her respondents (81 %) said that the Internet was the first thing they chose when they wanted to find health or healthcare information, but only 51% of them considered it a reliable source of information.

#### Generation Z (1995–2009)

Generation Z has got no experience of life before the Internet, technology was already accessible for them at a very young age (61). This generation is accustomed to interacting in a world that is connected all the time by means of advanced technology (e.g., tablet, smartphone, social media) (62).

Using focus group interviews Gray et al. (63) explored students' (between 11 and 19 years) perceptions and experiences of using the internet for seeking information about health and medicines. The internet was considered a primary general information source for this generation. They relied on radio and television alongside the Internet, which they preferred to books and leaflets. Adolescents perceived the internet as an alternative source of information for health problems and thought they might be able to avoid a visit to a health professional or be empowered from online information within the medical encounter.

College students with higher eHL are more likely to practice positive health behaviors (64). According to Stellefson et al. (65) students between 17 and 26 years often use the Internet to find health information and they feel comfortable using it. Nonetheless many of them have weak eHL skills related to searching for, retrieving, using and evaluating sources of eHealth information. Robb and Shellenbarger (66) state that college students (18–24 years) are able to retrieve health information on their own, but they are not confident enough about their knowledge to make decisions about health options independently. They are probably more reliant on their parents considering their health decisions.

#### Comparisons Alongside Age and Generations

Miller and Bell (32) examined the age differences in the role of trust and ease of search in predicting IHISB among four age groups (18–34, 35–49, 50–64, 65+). They concluded that the internet is a popular source of health information and that IHISB is negatively associated with age, with trust in the found information and with the perceived easiness of health information searches.

Aguilar-Palacio et al. (8) analyzed the micro- and macro factors affecting self-rated health and what role generational belonging plays in this relation. They divided their sample into four generations (silent generation—born before 1946, baby boomers, generation X and Y). They found that self-assessed health becomes worse with the aging of generations. Within the silent generation and the baby boomers, age was a more important factor, as for the self-assessed health of older individuals, it had an exponential effect.

Paige et al. (30) examined the attributes of the eHeals scale among Generation Y (18–35 years), X (36–51 years) and Baby boomers together with the Silent Generation (52–84 years). They proposed a 3-factor (information awareness, information seeking, information engagement) eHeals measurement model and concluded that it is valid for these age group comparisons. They found that older individuals have significantly lower eHeals score, smaller awareness of eHealth resources and less confidence in their information seeking and engagement skills on the Internet than younger people.

Magsamen-Conrad (7) investigated generational differences in new communication technology (NCT) use and eHL, among builders, boomers and generation X and Y. They found that builders had the fewest available resources and the lowest knowledge to use NCTs and the lowest eHL across all of the age groups. Baby boomers perceived to have more resources and knowledge about NCT use than builders but perceived less resources than the generation X.

Across different age-groups studies also provide empirical evidence for the negative association of HL and health care system utilization (37). The relationship between higher HL and less frequent use of health care services varies across countries (67), different patient groups (19) and it was dependent on the measured variable of the health service use (e.g., contacts to emergency services or hospital admissions vs. appointments at the GP or other health professionals). In the European HL project (67) long term health condition, self-perceived health status and gender predicted the frequency of visits by the doctor.

### Hypotheses

Our overall question is whether there are differences between IHISB, eHL (measured by eHeals) and empowerment across four generations in Hungary. Within this question we further focus on the relationships between these variables and certain health outcomes (self-rated health status, health care utilization) across the generations. The literature reviewed above enabled the formulation of the following six hypotheses:

We expect no generational differences in the use of the Internet for health purposes (68).

Following Paige et al.'s results (30) we hypothesize that older generations have lower eHeals score than younger ones.

We suppose that eHeals scores positively correlate with IHISB across all generations (17, 37).

In our fourth hypothesis we assume that the frequency of IHISB affects the utilization of the health care system in Generation X and Baby boomers, but eHeals scores do not (37, 67).

Good and bad subjective health status are associated with higher frequency of IHISB and higher eHeals scores across generations (10, 17), so we suggest a curvilinear relationship.

Following Robb and Shellenbarger's results (66), Generation Z got the least empowerment from using the Internet.

Our last assumption is that the frequency of IHISB and the eHeals score do not correlate with empowerment, but these variables determine together the measured health outcomes (subjective health status and the utilization of the healthcare system) (69, 70).

## Materials and Methods

### Sampling and Data Collection Procedure

Our cross-sectional study comprised collecting data from 522 subjects (155 male, 29.7%) belonging to four age cohorts in Hungary (Baby boomers, X, Y and Z generations), using a Web-based survey designed and tested in English-speaking countries (37). We aimed at having at least forty subjects in each age cohorts for group comparison, except in the group of Baby boomers, in which we aimed at least one hundred and fifty for comparing their data with the international ones. The data collection between 2018 January and June was carried out by part-time or full-time psychology students, who collected forty questionnaires each via convenience sampling in their own online environment as their course requirements. The subjects were asked to fill out an online 30-min-questionnaire about health-related issues. After having read an informed consent they agreed to participate by clicking a box in the first page of the online questionnaire. Further subjects were systematically selected between November 2018 and May 2020 by trying to make a more heterogeneous sample along gender and education. For doing this we used the Hungarian Statistical Office data regarding the Hungarian population in terms of proportion of gender and education. This phase took place—mainly by sending the link of the questionnaire online—in companies and retirement homes in Hungary. Ethical approval was obtained from the Psychology Ethical Committee of Universities in Hungary (111/2017). 11.9% (*N* = 62) of the sample possessed primary school education, 10.5% (*N* = 55) completed vocational school, 19.3% (*N* = 101) had a high-school graduation, 18.8% (*N* = 98) secondary grammar school education, and 38.9% (*N* = 203) graduated from college or university (Table 1 contains the sociodemographic characteristics of the sample).

**Table 1:** Socio-demographic characteristics of the sample (*N* = 522).

**Full sample size**		***N*** **=** **522**		
**Socio demographic characteristics**	**Variables**	**Number of participants**	**Percentage**	**Missing values**
Gender	Female	365	69.9%	2
	Male	155	29.7%	
Education	Primary school	62	11.9%	3
	Vocational school	55	10.5%	
	Secondary grammar school	101	19.3%	
	High school	98	18.8%	
	College/university	203	38.9%	
Generation (calculation is based on the variable “Year of birth”)	Generation Z	43	8.5%	16
	Generation Y	185	35.4%	
	Generation X	122	23.4%	
	Baby Boomers	156	29.9%	

### Measurements

Our main variables include IHISB, eHL (measured by eHeals), the self-perceived gain in empowerment by that information, and the number of health care appointments in the previous year. As we stated above—in a collaboration with Peter Schulz—we adopted an English test battery designed by Schulz et al. (37) to measure internet health behavior and health status of Anglo-saxon baby boomers.

We used a forward and back-translation procedure in order to create a conceptually equivalent Hungarian version of the test battery to the original English version. First, two English teachers, one of them is also a psychologist translated the items independently to Hungarian. Then a third independent bilingual person back-translated these to check for any inconsistencies. The final version of the test battery was designed by a professional group in health studies based on all the translations and the notes of the interpreters.

Internet health information seeking behavior was measured by 10 items describing different activities that are examples of Web-based information seeking, e.g., “I've looked online to try to diagnose a health condition,” “I've read or watched someone else's commentary or experience online about health-related issues.” The frequency of these behaviors was also asked using a 5-point scale ranging from “never” to “very often.” The 10 items were averaged to produce our variable of IHISB. The scale was found to be reliable (Cronbach alpha = 0.794, mean = 2.08, SD = 0.59, *N* = 263).

eHealth Literacy was measured by eHEALS (71), which comprises 8 items designed to measure awareness (items 1, 2), searching (items 3, 4), appraisal of health resources (items 6, 7), and utilization of electronic health information (items 5, 8). The scale is appropriate to measure self-reported ability to find, apprehend and use information on the internet as an indicator of the users' eHealth literacy. The items scored on a 5-point Likert-scale ranging from “strongly disagree” to “strongly agree.” Higher scores indicate greater self-reported skill. The scale was found to be reliable (Cronbach alpha = 0.94, mean = 29.54, SD = 7.217, *N* = 491).

Self-perceived gain in empowerment was measured by seven items designed by Schulz et al. (37). They covered self-perceived changes, e.g., In general, as a result of searching for health information online. “I feel more connected to others with a similar problem,” or “I can communicate more effectively with my health professional(s),” attributed to the use of the Internet. The items scored on a 5-point Likert scale ranging from “strongly disagree” to “strongly agree” with higher scores indicating higher self-perceived gain in empowerment. Using the measure produced reliable data (Cronbach alpha = 0.86, mean = 21.06, SD = 6.05, *N* = 489).

Our dependent variables were utilization of the health care system and self-rated health status. Unlike Schulz et al. (37) we not only measured the number of medical consultations with one's GP in the past 12 months, but also appointments with other health professionals, contacts to emergency services and hospital admissions. The number of visits was coded as 0, 1 time, 2 times, 3, 4, 5 to 9, and 10 or more times.

Self-Rated Health was measured by a single item: 1-bad/2-not too good/3-optimal/4-very good/5- excellent (24, 37).

Gender (male/female), age (year of birth), race (predefined categories e.g., Hungarian, Slovak or Roma identity), marital status (predefined categories e.g., I have never been legally married or registered in a civil union / I am a widow or widower or surviving civil union partner/I am legally married), educational attainment (predefined categories: Primary school/Vocational school/Secondary grammar school/High school/College or University), occupational status (predefined categories e.g., Employed full-time/Retired/Unemployed), income (open ended question: What was your total income from all sources before taxes last year) and the presence of chronic disease(s) (predefined categories e.g., None/Diabetes/Other) were self-reported by the participants (Table 1 contains the proportion of gender, generation (based on age) and education in the sample).

## Results

The statistical analysis of the data was performed using IBM SPSS for Windows 22 (72). One-way ANOVA was used for comparing the scores of the cohort-groups, and correlational and linear regression analysis was employed within the Baby boomer generation for further data analysis^1^. We agreed that the statistically significant *p*-value should be < 0.05.

### Hypotheses Testing

Our first hypothesis assumed that there are no differences among generations in the frequency of performing IHISB. Since our variable does not follow normal distribution (Kolmogorov-Smirnov test is 0.11), we used the Kruskall-Wallis test to compare the distribution in the four generations. We found no significant differences (p_IHISB_ = 0.54) (Table 2 contains the Means and Standard deviations of the variables in each generation), which supports our hypothesis.

**Table 2:** Means and Standard deviations of IHISB (a composite score of Internet Health Information Seeking Behavior) and eHEALS scores in generations of Baby boomers, X, Y, and Z.

**Generation**	**IHISB (missing values: 251)**			**eHEALS (missing values: 44)**		
	***N***	**M**	**SD**	***N***	**M**	**SD**
Baby Boomers	57	2.05	0.66	140	28.22	7.39
Generation X	72	2.06	0.53	117	29.7	7.31
Generation Y	110	2.11	0.62	180	30.93	6.8
Generation Z	32	2.14	0.53	41	29.15	5.36

In our second hypothesis we expected older generations to possess lower eHeals score than younger ones. This variable does not follow normal distribution in our sample (Kolmogorov-Smirnov test is 0.106), therefore we used the Kruskall-Wallis test to compare the distribution in the four generations. The difference was significant (*p* = 0.001) (Table 2 contains the Means and Standard deviations in each generation), so we ran the Dunn-Bonferroni rank-based *post-hoc* analysis, which indicated a significant difference (*p* = 0.001) between Baby Boomers and Generation Y in the expected direction.

We supposed that eHeals score positively correlated with IHISB across all generations. To test this hypothesis, first we used Spearman rank correlation in the whole sample, then within each generation. Significant associations were found in the whole sample [rho(265) = 0.25, *p* < 0.000] and in generation Y [rho(105) = 0.297, *p* = 0.002]. Both in the Baby boomer generation [rho(52) = 0.201, *p* = 0.145] and in generation Z [rho(29) = 0.241, *p* = 0.191] IHISB measured by the averaged frequency of certain related activities did not show correlation with eHeals, but eHeals had an association with the averaged frequency from whom (oneself, family, friend, colleague, health professionail, other) they search health information in the Internet [rho(62)_Boomers_ = 0.33, *p* = 0.008; rho(33)_GenerationZ_ = 0.405, *p* = 0.016]. In Generation X none of the variables correlated with each other. These results partially support our hypothesis.

In our fourth hypothesis we assumed that the averaged frequency of IHISB affects the utilization of the health care system in Generation X and Baby boomers, but eHeals score does not. First, we used Spearman rank correlation to test the associations of these variables. In the case of Baby boomers, IHISB showed a weak but significant correlation with regular health care utilization [rho(54) = 0.302, *p* = 0.024], but eHeals had no relation with it. In Generation X, however, we found the contradictory pattern: eHeals has a weak but significant correlation with health care use by appointments [rho(115) = 0.244, *p* = 0.08], but IHISB has not. In a linear regression model, eHeals affected health care utilization significantly (*R*^2^ = 0.06; Beta = 0.239; *p* = 0.009) in Generation X. These results partially support our hypothesis.

**Figure 1:**
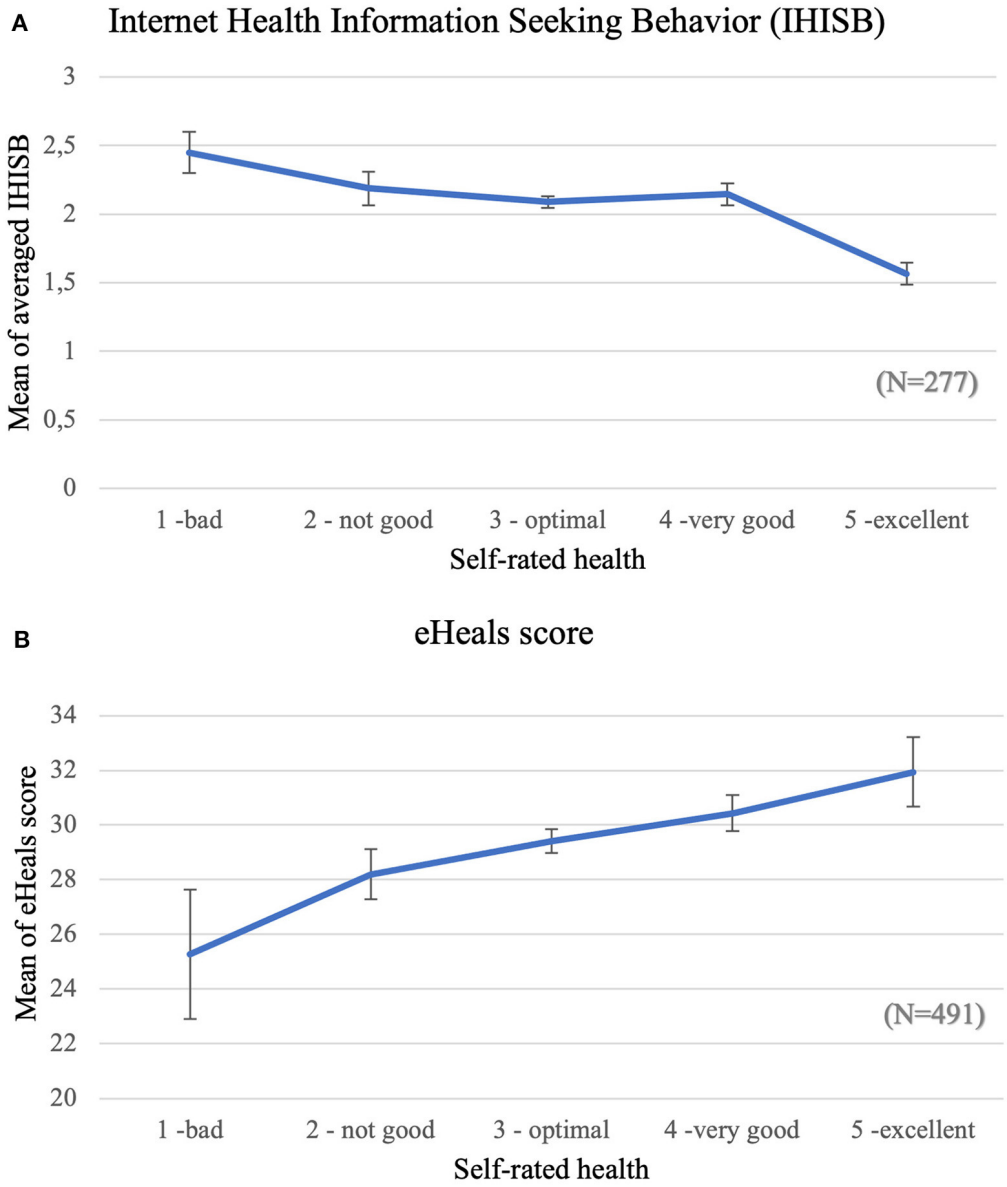
The distribution of **(A)** IHISB (a composite score of Internet Health Information Seeking Behavior) and **(B)** eHEALS values across self-rated health categories.

We hypothesized that the extreme values of subjective health status are associated with higher frequency of IHISB and higher eHeals in the whole sample. First, we used Spearman rank correlation to test this hypothesis, which showed significant but weak correlations between both subjective health status and IHISB [rho(175) = −0.138, *p* = 0.021] and subjective health status and eHeals [rho(489) = 0.164, *p* < 0.000] but in the opposite directions: while subjects who use the Internet more frequently to search for health information have worse self-rated health status, the ones with higher eHeals score report better subjective health status. Then we used the Kruskall-Wallis trial to test the distributions of IHISB and eHeals scores in each subjective health category. Both variables show significant differences alongside self-rated health status (p_IHISB_ = 0.001; p_eHEALS_ = 0.006) but not in the expected U-shape directions (see Figures 1A,B).

Beside self-rated health status, we also measured the presence of chronic disease with a question inquiring about 10 chronic diseases. Since we currently work on a project that deals with the association between HL and openness to new technologies among patients living with type-2 diabetes, we made some preliminary analyses comparing eHeals and IHISB alongside three groups: subjects without a chronic disease, diabetic patients and people living with a chronic illness other than diabetes. According to the Kruskall Wallis trial neither eHeals nor IHISB show difference between the three groups (*p* = 0.383, *p* = 0.067 respectively).

In our sixth hypothesis we assumed Generation Z gained the least empowerment from using the Internet. Since empowerment had a normal distribution in our sample (Kolmogorov-Smirnov test is 0.53, *p* = 0.06) we used One-way ANOVA to test this. It shows a continuous decrease in the score of empowerment across the generations from Boomers to Generation Z, and a significant difference between the generations [F_3_ = 3.23, *p* = 0.011], especially between Boomers and Generation Z (Bonferroni's *post hoc* test MD = 3.50, *p* = 0.006). (The difference between the other generations and generation Z was on the level of tendency.) This result supports our hypothesis.

Our last assumption was that IHISB and eHeals do not correlate with empowerment but these variables together will determine subjective health status and the utilization of the healthcare system. To test this, first we used Spearman rank correlation between IHISB, eHeals and empowerment. The results indicate significant moderate correlations between the variables: rho(271)_IHISB−empowerment_ = 0.54, *p* < 0.000 and rho(471)_EHEALS−empowerment_ = 0.414, *p* < 0.000. Then, we set up different linear regression models with the dependent variables, self-rated health status, using the health care system by appointment and using the health care system in emergency. The independent variables were IHISB, eHeals and empowerment. The result of the linear regression model (with Enter method) became significant in the following cases: self-rated health status is determined independently by eHeals (*R*^2^ = 0.023, Beta = 0.167, *p* = 0.001), but not by empowerment, visiting a doctor or a health-care professional by appointment is determined (*R*^2^ = 0.024, Beta = 0.153, *p* = 0.034) independently by empowerment but not IHISB, and using the health care system in emergency is both determined by eHeals negatively (*R*^2^ = 0.018, Beta = −0.108, *p* = 0.04) and empowerment (Beta = 0.145, *p* = 0.006). These do not support our original hypothesis, but give plausible results.

## Discussion

We aimed to explore generational differences in IHISB and digital HL (measured by eHeals) first in Hungary, as well as a self-rated and a more objective application of these skills (perceived empowerment and health care utilization). By involving a more complex social phenomenon (generation), a special context (Internet) and application in our research we have taken a further step into the direction of more contextualized, and at the same time more complex research of eHL.

Although Hack-Handa and Pinter (68) did not find it possible to compare Hungarian generations alongside IHISB due to the difficulties of reaching Generation Z and Baby Boomers representatively only via online platforms, we decided to use the more complex age-based category considering its significance in the attitude toward using technology (5–7) and in health status (8, 24).

In our first hypothesis we assumed no differences among generations in the frequency of producing IHISB, which was reported previously—mainly using age or age groups as variables—by Hungarian (17, 68) results, but not international (7, 10, 32) findings. Our findings support Hungarian results, since there were no differences in IHISB between the generations. It means that Hungarians between the age of 18 and 72 searched health information on the Internet equally frequently. Already in 2014, Tóth et al. (73) reported that in Hungary a significant majority of Internet users (87%) use the Internet to search health-related information, and we approached subjects who use the Internet for health purposes. Considering more recent data, Eurostat reported that in 2020, 63% of individuals used the internet for health information seeking in Hungary (11), which is above the average 55% of the 27 European countries. Our results detail this with the finding that the relatively high health-related Internet-usage does not differ between the generations.

In spite of the similar frequency of IHISB across the generations, the elder among them had less digital skills in finding information on the Web I (7, 30). The result of our second hypothesis also supported these international findings: there was a significant difference between Baby Boomers and Generation Y in the expected direction, i.e., the former generation had lower eHeals scores.

Although older generations have smaller awareness of eHealth resources and less confidence in their information seeking and engagement skills on the Internet than younger ones (30), college students (18–24 years) belonging to Generation Y and Z were not confident enough to make decisions about health options independently (66). In our sixth hypothesis we assumed and proved that empowerment gained by using the Internet decreased with age and was the lowest in Generation Z. This result is in accordance with the literature and can be crucial to plan health promotion programmes. It seems that younger generations need development in decision-making skills, while older ones need to be taught the effective use of the Internet. These shed further light on previous conclusions that highlighted older patients, who usually need the most medical attention, are the ones that lack the skills to use electronic health information and services effectively (26, 44, 74). However, the differences between the generations in eHeals scores can be interpreted in other ways as well. On the one hand, olders may face more complex situations, in which access to Internet-based information are more difficult and not so evident. This can result in lower eHeals scores. On the other hand, eHeals is a self-perceived assessment of health related digital skills, which means that digital natives may overestimate their competence in finding information on the Internet. To make clearer interpretation in future research eHL competence needs to be assessed.

The relationship between self-reported eHL skills (measured by eHeals) and IHISB seems to be more complicated if we look at it across generations (Hypothesis 3): only Generation Y showed a positive correlation between these variables. Baby boomers and Generation Z use their better self-reported eHL skills to search more health-information on the Web for others. While self-reported eHL skills and searching behavior did not associate with each other in Generation X. In the literature, Mitu (60) also reported that 18–31-year-old people (belonging mostly to Generation Y) with higher eHL produced more advanced IHISB (used more sources of information). Schulz et al. (37) found a moderate relationship between IHISB and eHeals in the Boomers generation, while Tennant et al.'s results (45) turned the attention toward differences within the older generations: younger age, more education, use of more electronic devices and the use of Web 2.0 platforms were associated with higher levels of eHL. In sum, it seems that more variety and frequency of IHISB might not be a sensitive variable in relation to the level of eHL skills, while being in relationship with others to search for can be a motivating factor for using eHL skills. We can use this latter explanation also in Generation Z: they are young enough not to deal extensively with their health, but if there is another person in their environment to search for health-related information on the Web, better eHL skills go along with more search. The technical ability of Generation X tends to be strong (49, 50) and in Hungary their overall health status is not very good. Taking these two into consideration, we can assume that this generation might search health-related information on the Web independently of their digital HL skills due to their needs and their belief that they are good in using this technology.

As outcome variables we used self-rated health status and the utilization of the healthcare system in their relation to IHISB, eHeals and empowerment. In our fourth hypothesis we assumed that in the case of Baby boomers and Generation X - when subjects need to focus on health problems - the utilization of the healthcare system is affected by IHISB, but not by eHeals score. According to our results in the case of Baby boomers IHISB showed a weak but significant correlation with regular health care utilization, but eHeals has no relation with it, which is in accordance with Schulz et al.'s (37) findings with path analysis. While in case of Generation X our results contradicted our expectations, because eHeals had a weak but significant correlation with health care use by appointments, and IHISB had not. We even could describe a causal effect from self-reported eHL to health care utilization in this generation. Seemingly, this positive relationship opposes not only Schulz et al.'s results (37), but also international ones that claim a negative association of HL and health care system utilization (37). In fact, our finding is in accordance with the literature that stresses the role of the measured variable of the health service use (67). The positive correlation in our sample was found between self-perceived eHL and the number of visits by a health professional by appointment. Conclusively, in Generation X digital skills (measured by eHeals) rather than Internet-seeking behavior affect the preventive, in-time interventive and regular maintaining visits to doctors. In this generation higher eHL might indicate higher awareness of health issues.

Regarding self-rated health status a U-shape relation was found between this outcome variable and IHISB and eHL in Hungary (17). Our results showed a different pattern: while subjects who use the Internet more frequently to search for health information have worse self-rated health status, the ones with higher self-perceived digital HL skills report better subjective health status. The latter relation is well-known between HL and subjective health status (75), and some international results also show that the higher the level of eHL is for an individual, the better self-rated health status he/she reports (22, 23).

Our last assumption was based on the Health Empowerment Model (HEM) (69, 70), which claims that HL and empowerment are different constructs, they do not correlate with each other, but they determine together certain health outcomes. We found significant moderate correlations both between IHISB and empowerment and eHeals and empowerment, which contradict our expectations. Self-rated health status was determined independently by eHeals, whereas visiting a health-care professional was predicted by empowerment. We can interpret it as doing something for our health needs empowerment. We revealed another determination: using the health care system in emergency is negatively determined by eHeals and positively by empowerment. So the ones who use healthcare services abruptly possess weaker self-reported eHL, but higher empowerment skills. They might belong to the category that HEM calls dangerous self-managers with low HL and high empowerment.

### Limitations

Altough we consider the generational approach as a strengh of our study, other scholars may find it an artificial theoretical construct. Other divisions of the age groups based on more detailed social and contextual information about the use of technology can be equally fruitful.

The main limitation of our study is the number of answers to the items. Although we collected a considerable number of responses, the degree of freedom varies heavily between statistical trials, because the instruction let the participants leave out sensitive questions.

Another limitation might be that we did not use validated instruments. However, the questionnaire was developed and used successfully previously in Anglo-saxon countries, and the reliability analyses showed good values of all the measurements in our sample.

Finally, although we used in the supplementary data collection phase the representative proportion of the Hungarian population in terms of gender and education that is provided by the Hungarian Statistical Office we did not manage to establish a representative sample. This lack of representativeness for the Hungarian population limits the generalizability of our results.

## Conclusion

We found using “generations” in digital health related topics more beneficial than age due to their common attitudes and skills toward technology and to their more similar health status and utilization of health care services. According to our knowledge our study is the first that focuses on generational differences in IHISB, self-perceived eHL (measured by eHeals) and related health outcomes in Hungary. Considering the Internet health information seeking the older generations (baby boomers and Generation x) shows the same frequency as the younger ones, which gives a solid motivation for developing their eHealth literacy skills. We find it crucial to plan the Hungarian health promotion programmes utilizing this high frequency of Internet health information seeking, since the eHealth literacy skills of older generations have an effect on their subjective health status, and gaining the relevant information regarding their health on the internet they are the most capable of applying it in making decisions. Our results also call the attention for the needs of Generation Z: to make better health decisions they need education in reflecting on the gained information and in applying it.

## Data Availability Statement

The raw data supporting the conclusions of this article are available from the corresponding author upon request.

## Ethics Statement

The studies involving human participants were reviewed and approved by Psychology Ethical Committee of Universities in Hungary (111/2017). The patients/participants provided their written informed consent to participate in this study.

## Author Contributions

OP-Z contributed to the conceptualization of the research process, organized the research process, performed data analysis, wrote the first draft, and finalized the final version of the manuscript. MH performed literature review and contributed to the writing of the first draft. PS and MC conceptualized the research process and critically reviewed and developed the manuscript. All authors contributed to the article and approved the submitted version.

## Conflict of Interest

The authors declare that the research was conducted in the absence of any commercial or financial relationships that could be construed as a potential conflict of interest.

## Footnotes

^1^The study materials and the details of all analyses are available from the corresponding author upon request.
